# Long noncoding RNA CASC11 suppresses sorafenib-triggered ferroptosis via stabilizing SLC7A11 mRNA in hepatocellular carcinoma cells

**DOI:** 10.1007/s12672-023-00761-9

**Published:** 2023-08-08

**Authors:** Fei Chen, Liang Wang

**Affiliations:** 1 Department of Ultrasound, The First Affiliated Hospital of Jinzhou Medical University, Jinzhou, China; 2 Department of General Surgery, The First Affiliated Hospital of Jinzhou Medical University, Jinzhou, 121001 Liaoning Province China

**Keywords:** SLC7A11, mRNA stability, Sorafenib, Ferroptosis

## Abstract

As a frontline treatment for patients with advanced hepatocellular carcinoma (HCC), sorafenib is an effective drug approved by the Food and Drug Administration (FDA). Ferroptosis, a newly defined programmed cell death process with the hallmark of the accumulation of iron-dependent lipid peroxides, can be induced by sorafenib treatment. Our previous study identified oncogenic roles of long noncoding RNA (lncRNA) Cancer susceptibility candidate 11 (CASC11) in HCC progression. However, the relationship between CASC11 and sorafenib-induced ferroptosis in HCC remains unclear. In the present study, we aim to investigate the role of CASC11 in sorafenib-induced ferroptosis in HCC cell lines and determine the involved molecular mechanisms. Here, we demonstrated that sorafenib decreased CASCL11 expression. Knockdown of CASC11 enhanced sorafenib-induced ferroptosis, while overexpression of CASC11 exerted the opposite effect in HCC cells. Moreover, CASC11 led to the accumulation of intracellular malondialdehyde (MDA), lipid reactive oxygen species (ROS) and Fe^2+^ while depleting glutathione (GSH), thereby suppressing sorafenib-induced ferroptosis and cell death. Ferrostatin-1 (Ferr-1), a ferroptosis inhibitor, reversed the enhanced anticancer effect of sorafenib caused by the silence of CASC11 in HCC cells. Mechanistically, CASC11 upregulated the expression of solute carrier family 7 member 11 (SLC7A11) which is critical for ferroptosis inhibition. CASC11 associated with and stabilized SLC7A11 mRNA. In summary, our data revealed, for the first time, that CASC11 inhibits the sorafenib-induced ferroptosis in HCC cells via regulating SLC7A11, providing a new basis for clinical therapeutic strategies for patients with HCC.

## Introduction

Hepatocellular carcinoma (HCC) is increasingly becoming a pressing public health issue, ranking as the third leading cause of cancer-related fatalities with a five-year survival rate of only 18% [1]. For advanced HCC patients who are not eligible for surgical resection, sorafenib is the first systemic therapy approved by the Food and Drug Administration (FDA). As compared to the placebo group, sorafenib improved the time to progression and extended overall survival by approximately 2.3–2.8 months [2, 3]. However, the existence of primary and acquired sorafenib resistance has limited the survival benefit.

Ferroptosis is a recently acknowledged form of regulated cell death that has been identified in numerous physiological and pathological conditions. Ferroptosis is characterized by abnormal accumulations of lipid hydroperoxides and lipophilic reactive oxygen species (ROS) in cellular membranes [4, 5]. The significance of ferroptosis in cancer has recently gained attention, as cancer cells have a greater demand for iron than normal cells, making them more susceptible to ferroptosis. In fact, ferroptosis can be induced in several cancer cell lines and xenograft models to inhibit tumor growth, drug resistance, and radioresistance [6, 7]. As a result, ferroptosis offers the potential for HCC therapy. Interestingly, sorafenib was identified as a potent inducer of ferroptosis [8]. The combination of ferroptosis inducer erastin and sorafenib remarkably enhances the anticancer effect of sorafenib [9]. Recent studies demonstrated that FNDC5, HBXIP and MT-1G play important roles in preventing ferroptosis and inducing sorafenib resistance in HCC cells [10–12]. Further exploration of the molecular mechanisms underlying the efficacy of sorafenib in HCC through ferroptosis may offer novel therapeutic approaches for the treatment of this disease.

Long non-coding RNAs are a class of non-coding RNAs that consist of more than 200 nucleotides. LncRNAs are dynamically expressed and play diverse roles in physiological and pathological processes via diverse mechanisms. For instance, lncRNAs interact with chromatin-modifying complexes or transcriptional factors to active or suppress gene transcription. They can regulate the stability of target mRNAs by associating with them. Moreover, lncRNAs indirectly modulate mRNA expression by regulating the activity of miRNA in mRNA binding. Additionally, lncRNAs are involved in the posttranslational modification of protein [13–15]. Recently, some lncRNAs have been found to regulate ferroptosis in cancer cells. For example, lncRNA HEPFAL, which is reduced in HCC tissues, promotes ferroptosis and induces the accumulation of lipid ROS and iron by inhibiting the stability of solute carrier family 7 member 11 (SLC7A11) [16]. LncRNA NEAT1 upregulates the expression of MIOX by competitively binding to miR-362-3p, which increases ROS production, decreases the intracellular glutathione (GSH) levels and enhances ferroptosis in HCC cells [17]. The long non-coding RNA, cancer susceptibility candidate 11 (CASC11), is a recently discovered molecule that has been shown to be upregulated in multiple human cancers. It plays a critical role in regulating various cellular processes, including proliferation, apoptosis, and metastasis of tumor cells [18–20]. Our previous study reported that CASC11 associates with and stabilizes Ubiquitin-conjugating enzyme E2T (UBE2T) mRNA, enhancing HCC growth and metastasis both in vitro and in vivo [21]. However, the role of CASC11 in regulating ferroptosis in HCC remains unclear. The objective of our current research is to explore the function and underlying mechanism of CASC11 in modulating ferroptosis and the anticancer efficacy of sorafenib in HCC cell lines. Our research suggests a novel link between CASC11 and ferroptosis.

## Materials and methods

### Cell culture

HCC cell lines, Hep3B and Huh7, were obtained from the Institute of Biochemistry and Cell Biology of the Chinese Academy of Sciences (Shanghai, China) and cultured in DMEM medium (Gibco) with 10% fetal bovine serum (Gibco) and 1% penicillin–streptomycin at 37 °C with 5% CO_2_.

### Tissue samples

HCC tissues were collected from 40 patients who were diagnosed with HCC at the First Affiliated Hospital of Jinzhou Medical University. Out of the 40 patients included in the study, 20 exhibited resistance to sorafenib treatment, while the remaining patients responded positively to sorafenib treatment. Prior to their participation in the study, all patients provided informed consent, and the research was conducted in accordance with the ethical principles outlined in the Declaration of Helsinki. The study was also approved by the Ethics Committee of the First Affiliated Hospital of Jinzhou Medical University.

### Plasmid transfection

Cells were transfected with empty vector or pCMV-SLC7A11 (GenePharma, Shanghai, China) using Turbofect Transfection Reagent (Thermo) according to the manufacturer’s instructions. 48 h later, the cells were used for further experiments.

### Construction of stable cells

The stable cells were constructed as our previous study described [21]. In brief, the lentiviral particles expressing scramble control or CASC11 shRNA (GenePharma, Shanghai, China) were infected into Hep3B cells, while Huh7 cells were infected with those expressing the empty vector or CASC11 or mutant CASC11 (GenePharma, Shanghai, China). The target sequences of shRNAs were shown as follows: scramble shRNA (shCon): TTCTCCGAACGTGTCACGT; shCASC11-1: TGCAGAAGGTCCGAAGAAA; shCASC11-2: GGTTCAGAGGTGACTATTC. The stable cells were selected for a period of two weeks using 2 μg/ml puromycin.

### Quantitative real-time polymerase chain reaction (qRT-PCR)

The total RNA from cells or tissues was extracted using Trizol reagent (Invitrogen) according to the standard protocol. For reverse transcription, PrimeScript™ 1st Strand cDNA Synthesis Kit (Takara) was used. Then, qRT-PCR detection was performed on ABI StepOne Plus System (Applied Biosystems) using a standard protocol from the SYBR® Green Premix Pro Taq HS qPCR Kit (Accurate Biology).

### Cell viability detection

Cell viability was measured using the Cell Counting Kit-8 (CCK-8) (Dojindo, Japan). 3.0 × 10^3^ cells were seeded into a 96-well plate. At various time points, 10 μl of CCK-8 reagent was added to each well and incubated for 1 h. Finally, the absorbance was measured at 450 nm using Multiskan™ FC System (Thermo Scientific).

### Colony formation assay

Cells were seeded into 6-well plates and treated with chemicals every 3 days for 14 days. Then, cells were fixed with paraformaldehyde and stained with crystal violet and then photographed.

### Western blot

Proteins were extracted by a RIPA lysis buffer (Beyotime Company, Beijing, China) containing protease inhibitor cocktail (Selleck) and quantified using a bicinchoninic acid (BCA) protein quantification kit (Thermo) according to manufacturer's instructions. Then, the western blot analysis was performed as per standard methods. The following antibodies were used: anti-SLC7A11 (Cell Signaling), anti-β-actin (Cell Signaling). The HRP-conjugated secondary antibodies were purchased from Jackson.

### RNA immunoprecipitation (RIP) and RNA pull-down assays

For detection of the interaction between CASC11 and SLC7A11 mRNA, MS2bs (MS2-binding protein)-MS2bp (MS2-binding sequences)-based RIP and RNA pull-down experiments were carried out as our previous study described [21]. Here, anti-GFP and negative control IgG antibodies were purchased from Abcam.

### Measurement of malondialdehyde (MDA)

Malondialdehyde (MDA) Colorimetric Assay Kit (Elabscience, Wuhan, China) was used to assess the MDA concentration according to the product instructions. The values of MDA were detected using a fluorescence microplate (Thermo) at 532 nm.

### Measurement of lipid ROS

The cellular ROS levels were measured using the Reactive Oxygen Species (ROS) Fluorometric Assay Kit (Elabscience, Wuhan, China) according to the product instructions. The values of ROS were detected by a fluorescence microplate (Thermo). The wavelength of excitation and emission was 500 nm and 525 nm, respectively.

### Measurement of ferrous iron (Fe^2+^) content

The level of intracellular Fe^2+^ was measured by using Cell Ferrous Iron Colorimetric Assay Kit (Elabscience, Wuhan, China) according to the manufacturer’s instructions. The absorbance at 593 nm was measured using a colorimetric microplate reader (Thermo).

### Measurement of glutathione (GSH)

The GSH concentration was analyzed using the Reduced Glutathione (GSH) Colorimetric Assay Kit (Elabscience, Wuhan, China) according to the product instructions. The absorbance at 405 nm was measured using a colorimetric microplate reader (Thermo).

### Cystine uptake assay

Cells were seeded in a 12-well plate and incubated with DMEM (which contains 200 µM cystine) containing [^14^C] cystine (PerkinElmer) (0.04 μCi) for the indicated time periods. Upon termination of uptake, cells were rinsed twice in cold PBS and then lysed in 0.1 mM NaOH. With a quench curve present, radioactivity (DPM) was measured using a Tri-Carb® Liquid Scintillation Analyzer (PerKinElmer).

### Luciferase reporter assay

Full-length transcript of SLC7A11 was cloned and incorporated into the pmirGLO plasmid (Promega). Reporter plasmids for SLC7A11 were transfected into the cells. Following a 48-h incubation period, the cells were analyzed using the Dual-Luciferase™ Reporter (DLR™) Assay Systems. The luciferase activity was quantified and determined as the ratio of firefly luciferase activity to renilla luciferase activity.

### Statistical analyses

Statistical analysis was conducted using SPSS 16.0. Differences among different groups were analyzed by the two-tailed student’s t test or one-way ANOVA followed by Bonferroni test for multiple comparison. The association between CASC11 and SLC7A11 mRNA expression was evaluated through Pearson correlation analysis. Statistically significant difference between groups was considered at P < 0.05.

## Results

### Sorafenib treatment decreases CASC11 expression in HCC cells

**Fig. 1:**
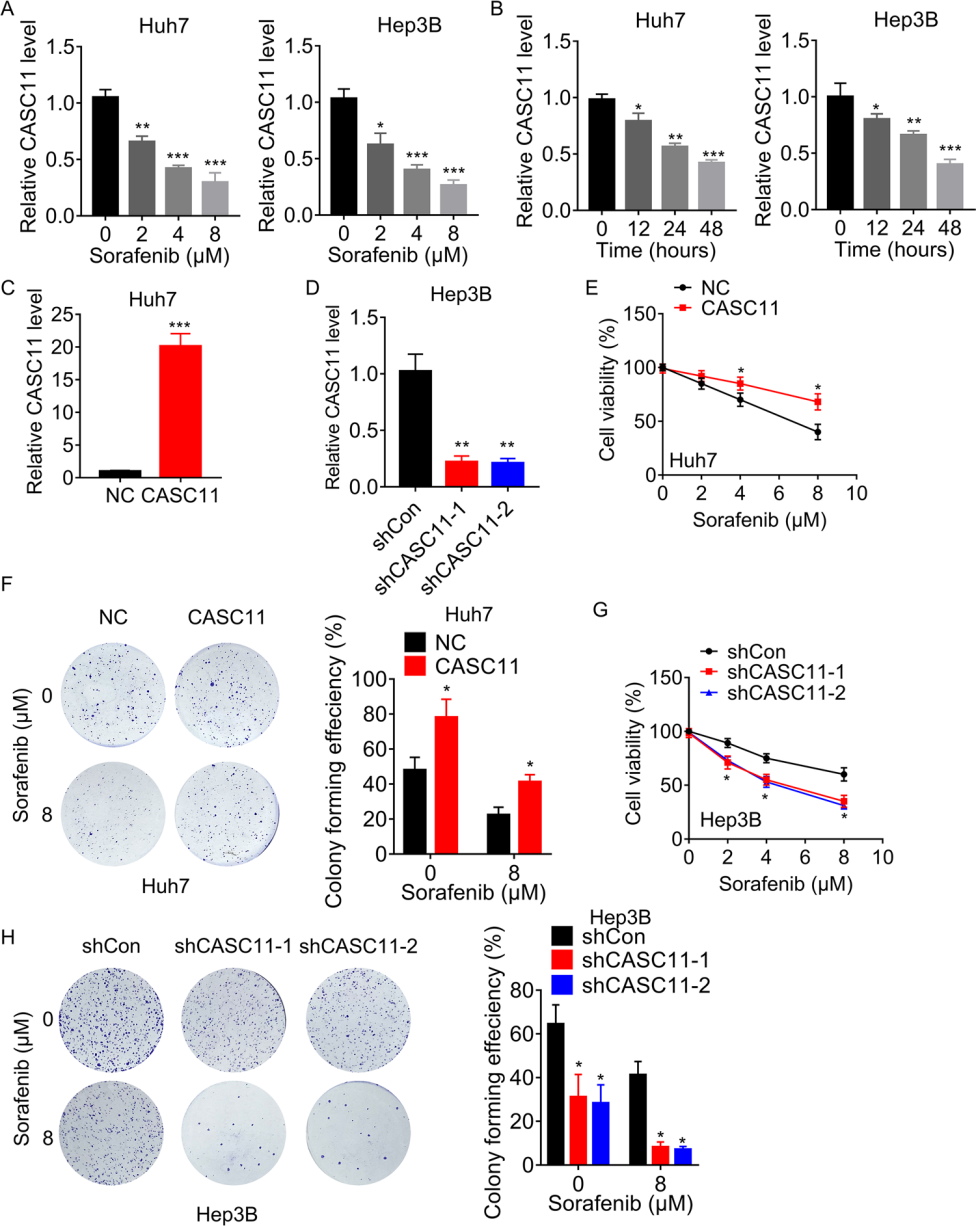
CASC11 is decreased by sorafenib in HCC cells. **A** Huh7 and Hep3B cells were treated with different concentration of sorafenib for 48 h, then the CASC11 levels were detected using qRT-PCR. **B** Huh7 and Hep3B cells were treated with 8 μM sorafenib for different time, then the CASC11 levels were detected using qRT-PCR. **C** CASC11 was overexpressed in Huh7 cells. The overexpression efficiency was validated using qRT-PCR. **D** CASC11 was knocked down in Hep3B cells. The knockdown efficiency was validated using qRT-PCR. **E** Cell viability was measured in control and CASC11-overexpressing Huh7 cells treated with indicated concentration of sorafenib for 48 h. **F** Colony formation of control and CASC11-overexpressing Huh7 cells treated with sorafenib. **G** Cell viability was measured in control and CASC11-silencing Hep3B cells treated with indicated concentration of sorafenib for 48 h. **H** Colony formation of control and CASC11-silencing Hep3B cells treated with sorafenib. *p < 0.05; **p < 0.01; ***p < 0.001

First, we investigated whether sorafenib regulates the expression of lncRNA CASC11. Hep3B and Huh7 cells were treated with different concentration of sorafenib, then the CASC11 expression was measured using qRT-PCR. Our results indicated a significant decrease in CASC11 expression in a dose- and time-dependent manner upon sorafenib treatment (Fig. 1A, B).

According to the endogenous expression levels of CASC11 in different HCC cell lines from our previous study [21], we established Hep3B cells with stable CASC11 knockdown and Huh7 cells with stable CASC11 overexpression, respectively (Fig. 1C, D). Subsequently, whether CASC11 affects the suppressive effect of sorafenib on HCC cell survival was examined. As shown by the results of CCK-8 and colony formation assays, CASC11 overexpression in Huh7 cells rendered cells more resistant to sorafenib-induced cell death (Fig. 1E, F). Conversely, sorafenib treatment significantly reduced cellular viability, which was further strengthened in CASC11-silencing Hep3B cells (Fig. 1G, H). These findings indicated that CASC11 can be downregulated by sorafenib treatment, and CASC11 attenuates the inhibitory efficiency of sorafenib in HCC cells.

### CASC11 inhibits ferroptosis in sorafenib-treated HCC cells

**Fig. 2:**
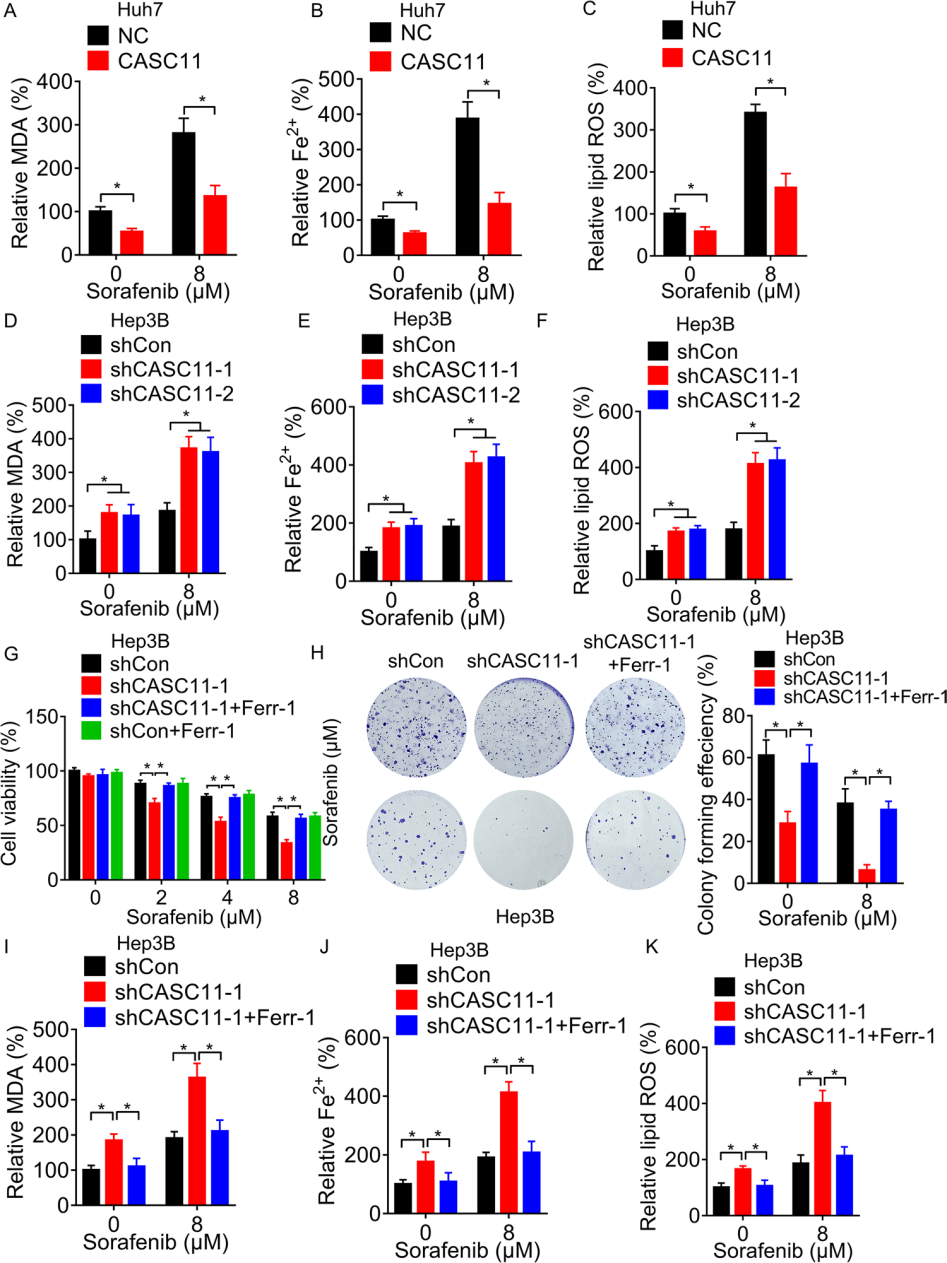
CASC11 inhibits ferroptosis in sorafenib-treated HCC cells. **A** The intracellular MDA levels were measured in control and CASC11-overexpressing Huh7 cells treated with 0 or 8 µM sorafenib for 48 h. **B** The intracellular Fe^2+^ levels were measured in control and CASC11-overexpressing Huh7 cells treated with 0 or 8 µM sorafenib for 48 h. **C** The intracellular lipid ROS levels were measured in control and CASC11-overexpressing Huh7 cells treated with 0 or 8 µM sorafenib for 48 h. **D** The intracellular MDA levels were measured in control and CASC11-knockdown Hep3B cells treated with 0 or 8 µM sorafenib for 48 h. **E** The intracellular Fe^2+^ levels were measured in control and CASC11-knockdown Hep3B cells treated with 0 or 8 µM sorafenib for 48 h. **F** The intracellular lipid ROS levels were measured in control and CASC11-knockdown Hep3B cells treated with 0 or 8 µM sorafenib for 48 h. **G** CCK-8 assay was performed to test the cell viability of control and CASC11-knockdown Hep3B cells treated with the gradient concentrations of sorafenib. 10 μM ferrostatin-1 (a ferroptosis inhibitor, Ferr-1) or DMSO along with sorafenib was added into shCon and shCASC11 group. **H** A colony formation assay of control and CASC11-knockdown Hep3B cells treated with 0 or 8 µM sorafenib for 2 weeks. 10 μM Ferr-1 or DMSO along with sorafenib was added into shCASC11 group. **I** The intracellular MDA levels were measured in control and CASC11-knockdown Hep3B cells treated with 0 or 8 µM sorafenib for 48 h. 10 μM Ferr-1 or DMSO along with sorafenib was added into shCASC11 group. **J** The intracellular Fe^2+^ levels were measured in control and CASC11-knockdown Hep3B cells treated with 0 or 8 µM sorafenib for 48 h. 10 μM Ferr-1 or DMSO along with sorafenib was added into shCASC11 group. **K** The intracellular lipid ROS levels were measured in control and CASC11-knockdown Hep3B cells treated with 0 or 8 µM sorafenib for 48 h. 10 μM Ferr-1 or DMSO along with sorafenib was added into shCASC11 group. *p < 0.05; **p < 0.01; ***p < 0.001

As ferroptosis, rather than apoptosis, is the key mechanism underlying the cell death induced by sorafenib, we aimed to investigate the possible involvement of CASC11 in this process. Fe^2+^ and lipid ROS are essential for ferroptosis process, and malondialdehyde (MDA) is an important end product of lipid ROS [22]. Elevated levels of MDA, ROS, and Fe^2+^ are indicative of ferroptosis. We found that upregulation of CASC11 decreased the levels of MDA, ROS and Fe^2+^ (Fig. 2A–C), whereas depletion of CASC11 increased them (Fig. 2D–F). Moreover, in the context of sorafenib treatment, knockdown of CASC11 also led to the obvious accumulation of intracellular MDA, lipid ROS and Fe^2+^ in Hep3B cells (Fig. 2D–F), while overexpression of CASC11 reduced their concentrations in Huh7 cells (Fig. 2A–C).

We explored whether CASC11 could mitigate sorafenib-induced cell death via ferroptosis inhibition. Treatment with ferrostatin-1 (Ferr-1), a ferroptosis inhibitor, reversed the cell death induced by sorafenib. The rate of cell death induced by sorafenib following CASC11 knockdown in the Ferr-1 group was much lower than that in the group without Ferr-1, but it did not differ significantly from that in the shCon + Ferr-1 group (Fig. 2G). This finding was further validated by a colony formation assay (Fig. 2H). Moreover, the increase of MDA, Fe^2+^ and lipid ROS could be blocked by Ferr-1 in CASC11-knockdown Hep3B cells (Fig. 2I–K). Taken together, these results suggested that ferroptosis is inhibited by CASC11, attenuating sorafenib-induced the cell death.

### CASC11 associates with and stabilizes SLC7A11 mRNA

**Fig. 3:**
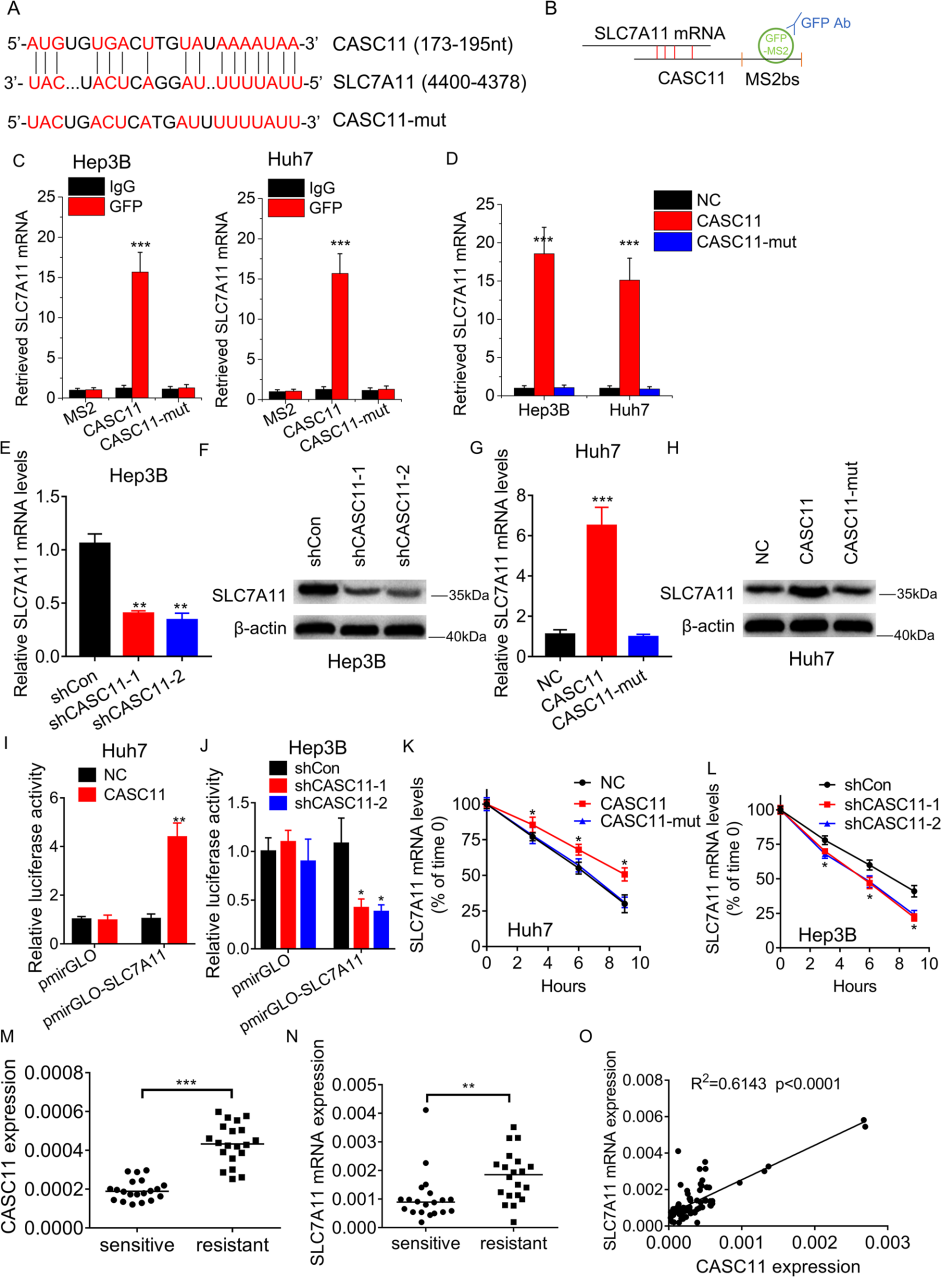
CASC11 stabilizes SLC7A11 mRNA. **A** BLAST analysis was used to identify the region of high complementarity between CASC11 and SLC7A11 mRNA. **B** The schematic diagram of MS2bs-MS2bp-based RIP (MS2-RIP) experiment. **C** The MS2-RIP followed by qRT-PCR was carried out to validate the interaction between CASC11 and SLC7A11 mRNA. **D** Cell lysates were incubated with biotin-labeled CASC11; after pull-down, mRNA was extracted and assessed by qRT-PCR. **E** The SLC7A11 mRNA levels were measured by qRT-PCR in control and CASC11-silencing Hep3B cells. **F** The SLC7A11 protein levels were measured by western blot in control and CASC11-silencing Hep3B cells. **G** The SLC7A11 mRNA levels were measured by qRT-PCR in control and wild-type or mutant CASC11-overexpressing Huh7 cells. **H** The SLC7A11 protein levels were measured by western blot in control and wild-type or mutant CASC11-overexpressing Huh7 cells. **I** The pmirGLO or pmirGLO-SLC7A11 was transfected into control and CASC11-overexpressing Huh7 cells. 48 h later, the luciferase activity was tested. Data are presented as the relative ratio of firefly luciferase activity to renilla luciferase activity. **J** The pmirGLO or pmirGLO-SLC7A11 was transfected into control and CASC11-silencing Hep3B cells. 48 h later, the luciferase activity was tested. Data are presented as the relative ratio of firefly luciferase activity to renilla luciferase activity. **K** Control = and wild-type or mutant CASC11-overexpressing Huh7 cells were treated with Actinomycin D (5 μg/mL). At different time point, the loss of SLC7A11 mRNA was detected using qRT-PCR. **L** Control and CASC11-silencing Hep3B cells were treated with Actinomycin D (5 μg/mL). At different time point, the loss of SLC7A11 mRNA was detected using qRT-PCR. **M** The expression of CASC11 was examined using qRT-PCR in HCC tissues from 20 patients with sorafenib resistance and 20 patients with sorafenib sensitivity. **N** The expression of SLC7A11 mRNA was examined using qRT-PCR in HCC tissues from 20 patients with sorafenib resistance and 20 patients with sorafenib sensitivity. **O** Correlation analysis between CASC11 and SLC7A11 expression in HCC tissues. *p < 0.05; **p < 0.01; ***p < 0.001

Then, the underlying mechanism by which CASC11 regulates ferroptosis in HCC cells was investigated. Our previous study identified the target mRNA associated with CASC11 using a MS2bs-MS2bp-based RIP experiment followed by RNA-sequencing [8]. Interestingly, we found that SLC7A11 mRNA may interact with CASC11. As an essential part of the system Xc-, SLC7A11 is an important regulator of ferroptosis. SLC7A11 has been confirmed to be overexpressed in some kinds of cancer and attenuate ferroptosis [23]. Therefore, we hypothesized that SLC7A11 might act as a downstream mediator of CASC11 in the regulation of ferroptosis. BLAST analysis (http://blast.ncbi.nlm.nih.gov/) was used to identify the region of high complementarity between CASC11 and SLC7A11 mRNA. (Fig. 3A). We created a mutation in the binding sites of CASC11 (named CASC11-Mut) with SLC7A11 mRNA and performed an MS2bs-MS2bp-based RIP assay to confirm CASC11's direct interaction with SLC7A11 mRNA (Fig. 3B). In both Huh7 and Hep3B cells, CASC11 significantly enriched SLC7A11 mRNA compared to empty vectors, IgG, and CASC11-Mut (Fig. 3C). An RNA pull-down assay further confirmed the interaction of CASC11 with SLC7A11 mRNA (Fig. 3D). Moreover, the results of western blot and qRT-PCR experiments revealed that knockdown of CASC11 significantly reduced the mRNA and protein levels of SLC7A11 in Hep3B cells (Fig. 3E, F). In Huh7 cells, wild-type CASC11, but not CASC11-Mut, was found to upregulate the expression of SLC7A11 (Fig. 3G, H). The luciferase reporter containing SLC7A11 was constructed (pmirGLO-SLC7A11). CASC11 overexpression increased the luciferase activity of pmirGLO-SLC7A11 (Fig. 3I), while knockdown of CASC11 decreased this (Fig. 3J). In order to determine whether CASC11 regulates SLC7A11 mRNA stability, cells with CASC11 knockdown or overexpression were treated with Actinomycin D, an inhibitor of RNA synthesis. The loss of SLC7A11 mRNA was monitored at different time points using qRT-PCR. The overexpression of wild-type CASC11, but not its mutant, effectively inhibited the degradation of SLC7A11 mRNA (Fig. 3K). Conversely, the knockdown of CASC11 was observed to reduce the half-life of SLC7A11 mRNA (Fig. 3L).

To further validate the regulatory relationship between CASC11 and SLC7A11, a qRT-PCR assay was carried out to examine their expression in HCC tissues. It was shown that HCC tissues obtained from patients with sorafenib resistance exhibited elevated levels of CASC11 and SLC7A11 expression, in comparison to those obtained from patients with sorafenib sensitivity (Fig. 3M, N). We also observed a positive correlation between CASC11 and SLC7A11 expression (Fig. 3O). Collectively, our data suggested that CASC11 associates with SLC7A11 mRNA and inhibits its degradation.

### CASC11 enhances cystine uptake and increases GSH level via SLC7A11

**Fig. 4:**
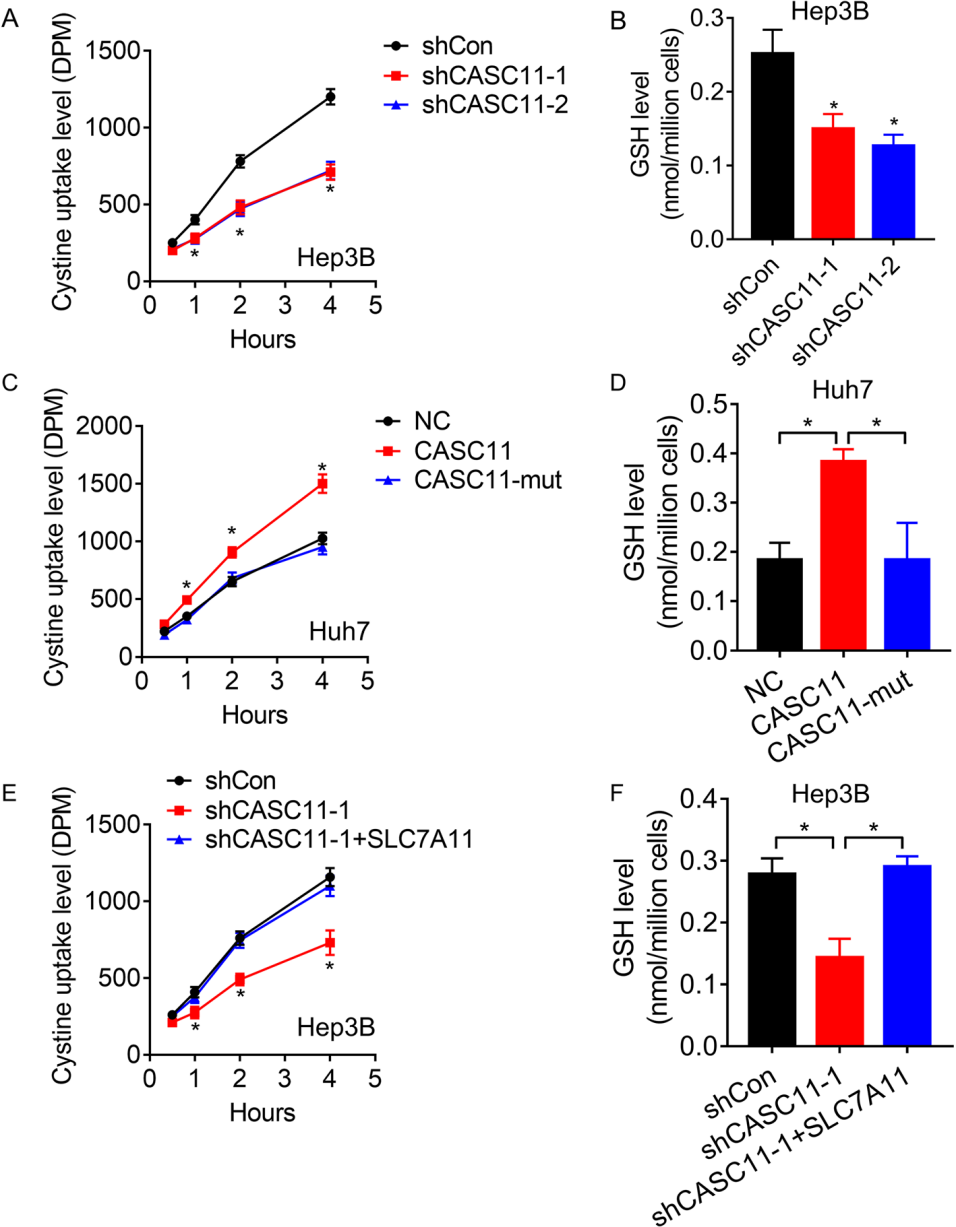
CASC11 enhances cystine uptake and increased GSH levels. **A** Cystine uptake levels were measured in control and CASC11-silencing Hep3B cells. **B** Bar graph showing intracellular GSH levels in control and CASC11-silencing Hep3B cells. **C** Cystine uptake levels were measured in control and CASC11-overexpressing Huh7 cells. **D** Bar graph showing intracellular GSH levels in control and CASC11-overexpressing Huh7 cells. **E** Cystine uptake levels were measured in CASC11-silencing Hep3B cells transfected with SLC7A11. **F** Bar graph showing intracellular GSH levels in CASC11-silencing Hep3B cells transfected with SLC7A11. *p < 0.05

SLC7A11 is mainly responsible for absorbing extracellular cystine, and cystine is used by cells to produce glutathione (GSH), a reducing substance. GSH is utilized by GPX4 to detoxify lipid hydroperoxide and to protect cells from ferroptosis [24]. Therefore, the cystine uptake and GSH level in the HCC cells was measured. The results indicated that the inhibition of cystine uptake and reduction of GSH level were observed upon the knockdown of CASC11 in Hep3B cells (Fig. 4B). The overexpression of wild-type CASC11, but not its mutant, resulted in the enhancement of cystine uptake and increase of GSH level in Huh7 cells (Fig. 4C, D). Notably, restoration of SLC7A11 abolished the decrease in cystine uptake and GSH levels caused by CASC11 depletion (Fig. 4E, F). These findings suggested that CASC11 regulates cystine uptake and GSH synthesis via SLC7A11.

### CASC11 regulates ferroptosis via SLC7A11 to affect the efficacy of sorafenib

**Fig. 5:**
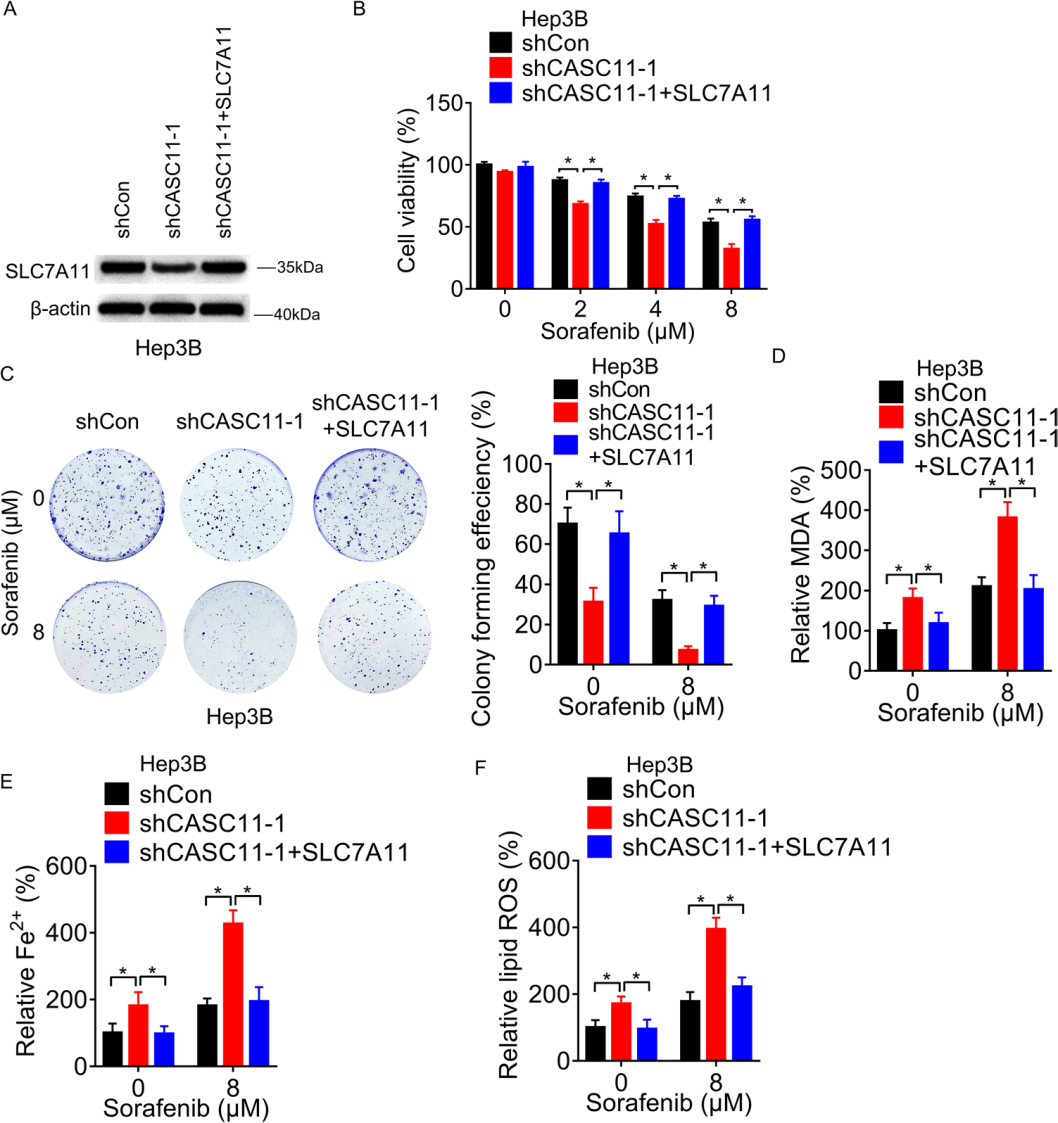
CASC11 regulates ferroptosis by modulating the expression of SLC7A11. **A** SLC7A11 was overexpressed in CASC11-silencing Hep3B cells, then western blot assay was performed. **B** Cell viability was measured by CCK8 assays in CASC11-silencing Hep3B cells transfected with SLC7A11 and treated with 0 or 8 µM sorafenib. **C** Colony formation assay of CASC11-silencing Hep3B cells transfected with SLC7A11 and treated with 0 or 8 µM sorafenib. **D** The concentration of MDA in CASC11-silencing Hep3B cells transfected with SLC7A11 and treated with 0 or 8 µM sorafenib for 48 h. **E** The concentration of intracellular Fe^2+^ in CASC11-silencing Hep3B cells transfected with SLC7A11 and treated with 0 or 8 µM sorafenib for 48 h. **F** The concentration of intracellular lipid ROS in CASC11-silencing Hep3B cells transfected with SLC7A11 and treated with 0 or 8 µM sorafenib for 48 h. *p < 0.05

To investigate whether CASC11-SLC7A11 axis is critical for repressing sorafenib-induced ferroptosis, CASC11-knockdown Hep3B cells were transfected with SLC7A11 plasmid (Fig. 5A). Firstly, the CCK-8 assay was performed to detect the cell survival. Sorafenib inhibited the proliferation of Hep3B cells, which was further enhanced by CASC11 knockdown. Moreover, overexpression of SLC7A11 restored the inhibitory effect of sorafenib (Fig. 5B). Consistent with the above findings, the colony formation assay revealed that the augmented potency of sorafenib resulting from CASC11 knockdown was nullified by the upregulation of SLC7A11 (Fig. 5C).

To further clarify the significance of the CASC11-SLC7A11 axis in sorafenib-mediated ferroptosis, the accumulation of intracellular MDA, lipid ROS and Fe^2+^ was measured. Remarkably, the overexpression of SLC7A11 mitigated the sorafenib-induced accumulation of intracellular MDA, lipid ROS, and Fe^2+^ in Hep3B cells with silenced CASC11 expression (Fig. 5D, F). Collectively, our findings suggested that CASC11 promotes resistance to sorafenib-induced ferroptosis in HCC cells by upregulating the expression of SLC7A11.

## Discussion

The process of transcription and translation is dynamically controlled by long noncoding RNAs, which are closely linked to cancer development. Among them, CASC11 has been found to function as an oncogenic lncRNA in various kinds of human cancers, including colorectal cancer, gastric cancer, bladder cancer, ovarian cancer, and HCC. CASC11 facilitates proliferation, metastasis, tumorigenesis, and chemoresistance [18, 21, 25–28]. We previously demonstrated that CASC11 is overexpressed in HCC tissues and indicates poor prognosis of HCC patients [21]. However, to date, the impact of CASC11 on the ferroptosis of HCC cells remains ambiguous. Here, we reported that lncRNA CASC11 exerts regulatory control over ferroptosis in HCC cells by acting upstream of the critical ferroptosis-associated gene SLC7A11. In vitro experiments demonstrated that CASC11 impeded the sorafenib-induced ferroptosis. Correspondingly, CASC11 curbed the accumulation of lipid ROS, MDA and Fe^2+^ in HCC cells. Further in vivo assays are needed to provide more profound evidence of the suppressive effect of CASC11 on ferroptosis. Apart from CASC11, recent studies have identified other ferroptosis-related lncRNAs in HCC. For example, lncRNA HEPFAL promotes ferroptosis by enhancing the degradation of SLC7A11 protein and elevating the levels of lipid ROS and iron [16]. Lowering the expression of lncRNA LINC01134 expression enhances the accumulation of lipid ROS and MDA, while reducing the GSH/GSSG ratio, ultimately leading to ferroptosis through GPX4 [29]. Moreover, lncRNA NEAT1 enhances ROS production by upregulating MIOX expression, which in turn decreases intracellular NADPH and GSH levels, resulting in intensified ferroptosis [17]. Altogether, these findings highlight the critical role of lncRNAs in regulating ferroptosis in HCC.

Normal cells use system Xc- as a cysteine/glutamate transporter to transport cysteine into the cell and exchange it for glutamate. SLC7A11 is an important part of system Xc-. The expression of SLC7A11 was found to be a prognostic indicator for the survival of HCC patient, as high levels of SLC7A11 expression are significantly associated with unfavorable clinical outcomes [30]. Cystine taken up into cells by SLC7A11 is converted into cysteine by a reduction reaction that consumes NADPH, and cysteine is used to synthesize GSH [31, 32]. Cancer cells require cysteine to maintain redox balance and survive. Tumor cells die from ferroptotic cell death when cystine supply is insufficient [33]. During tumor growth, tumor cells upregulate the expression of SLC7A11 and maintain a high cystine uptake rate. However, there is still an incomplete understanding of how tumor cells maintain high levels of SLC7A11. A recent study reported that BAP1 inhibits cystine uptake and makes cells more susceptible to ferroptosis by deubiquitinating H2Aub on SLC7A11. Inactivation of BAP1 in tumor cells results in increased expression of SLC7A11, leading to enhanced cystine uptake, GSH synthesis, and resistance to ferroptosis [34]. Moreover, SLC7A11 expression is regulated post-translationally. For instance, SOCS2 serves as a bridge between ubiquitin and SLC7A11, promoting polyubiquitination degradation of SLC7A11, which leads to ferroptosis of HCC [35]. Here, we have uncovered a novel post-transcriptional regulatory mechanism for SLC7A11 expression. We identified a direct interaction between CASC11 and SLC7A11 mRNA, resulting in the stabilization of SLC7A11 mRNA. Our previous study has reported that CASC11 physically associates with UBE2T mRNA, suppressing its degradation via recruiting the RNA demethylase ALKBH5 and decreasing its m^6^A level [21]. Whether SLC7A11 mRNA is regulated by CASC11 in the same way needs further investigation.

## Conclusion

**Fig. 6:**
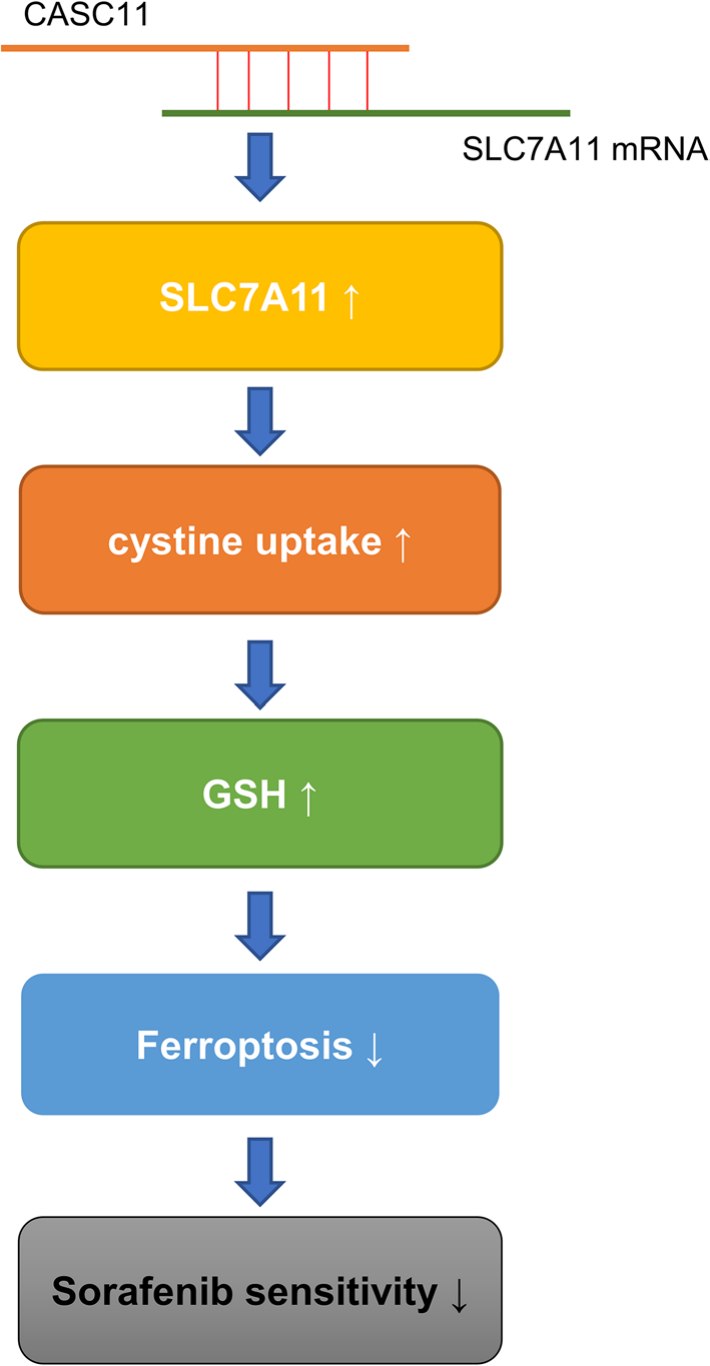
The schematic illustration of CASC11 regulating ferroptosis

To summarize, our research has demonstrated that the long non-coding RNA CASC11 enhances the stability of SLC7A11 mRNA, resulting in a decrease in the accumulation of lipid ROS, MDA, and Fe2+, and promoting the uptake of cystine and synthesis of GSH. This ultimately inhibits sorafenib-induced ferroptosis (Fig. 6). Given the clinical evidence of chemotherapy intolerance among HCC patients, ferroptosis induction may be a promising therapeutic strategy for HCC patients with high CASC11 levels.

## Data Availability

The datasets used during this research are available.

## Abbreviations

HCC: Hepatocellular carcinoma
CASC11: Long noncoding RNA cancer susceptibility candidate 11
CCK-8: Cell Counting Kit-8
SLC7A11: Solute carrier family 7 member 11
lncRNAs: Long noncoding RNAs
qRT-PCR: Quantitative real-time PCR
Ferr-1: Ferrostatin-1
ROS: Reactive oxygen species
